# Dynamics of decision-making: from evidence accumulation to preference and belief

**DOI:** 10.3389/fpsyg.2013.00758

**Published:** 2013-10-18

**Authors:** Marius Usher, Konstantinos Tsetsos, Erica C. Yu, David A. Lagnado

**Affiliations:** ^1^Department of Psychology and Sagol School of Neuroscience, Tel-Aviv UniversityRamat-Aviv, Israel; ^2^Department of Experimental Psychology, University of OxfordOxford, UK; ^3^Department of Psychology, University of MarylandMaryland, MD, USA; ^4^Department of Cognitive, Perceptual and Brain Science, University College LondonLondon, UK

**Keywords:** decision making, belief, evidence accumulation, data-generating process, problem solving

Decision-making is a dynamic process that begins with the accumulation of evidence and ends with the adjustment of belief. Each step is itself subject to a number of dynamic processes, such as planning, information search and evaluation. Furthermore, choice behavior reveals a number of challenging patterns, such as order effects and contextual preference reversal. Research in this field has converged toward a standard computational framework for the process of evidence integration and belief updating, based on sequential sampling models, which under some conditions are equivalent to normative Bayesian theory (Gold and Shadlen, 2007). A variety of models have been developed within the sequential sampling framework that can account for accuracy, response-time distributional data, and the speed-accuracy trade-off (Busemeyer and Townsend, 1993; Usher and Mcclelland, 2001; Brown and Heathcote, 2008; Ratcliff and McKoon, 2008). Yet there are differences between these models with regard to the mechanism of decision-termination, the optimality of the decision and the temporal weighting of the evidence. There is also a need to extend this framework to preference type of decisions (where the criteria are up to the judge) and to enrich it so as to include control processes (such as exploration/exploitation), information search, and adaptation to the environment, thereby allowing it to capture richer decision problems; for example, when alternatives are not pre-defined, or when the decision-maker is not just accumulating evidence but also adapting beliefs about the data-generating process.

This Research Topic presents new work that investigates the dynamical and mathematical properties of evidence integration and its neural mechanisms and extends this framework to more complex decisions, such as those that occur during risky choice, preference formation, and belief updating. We hope these articles will encourage researchers to explore the computational and normative aspects of the decision process and the observed deviations. We briefly review here the contributions in this collection, starting from simple perceptual decisions in which the information flow is externally controlled to more complex decisions, which allow the observer to control the information flow and other learning strategies, and following on with preference formation.

## Fast perceptual decisions

The first group of seven articles examines issues that arise in fast perceptual decisions that only allow the subject to control the weighting of the incoming evidence and the termination rule. Nevertheless, the integration time-scale, the temporal weights, and evidence termination can vary and this strongly affects the decision performance (how close people are to optimality) and the fit with the data. Some of these papers also examine the neural mechanisms that implement the decisions. In a mathematically-oriented paper Heathcote and Love (2012) examine a variant of a race model (the linear ballistic accumulator; LBA), which, under certain assumptions about the underlying distributions of starting point and drift-rate variability of evidence accumulation, allows for closed analytical formulas for the full response-time distribution in a lexical decision task and obtains a goodness of fit almost as good as that of the standard LBA model. In another formal paper, van Ravenzwaaij et al. (2012) examine, within the standard drift-diffusion model, the optimality of evidence accumulation strategies in decision situations with unequal frequency of stimuli types. They converge on the result that a bias in the decision starting point is optimal, in both fixed and variable difficulty conditions, though it appears that observers do not fully follow this strategy (but see Moran and Usher, in preparation). In another paper that examines sequential effects and decision biases in binary choice tasks, Goldfarb et al. (2012) present a simple extension of the standard decision model, which assumes changes in starting point depend on stimulus repetitions and alternations, combined with a response criteria increase following errors. This model accounts for a rich data of sequential dependence in response time and accuracy. In a paper contributed by Tsetsos et al. (2012), the aim was to contrast the standard drift-diffusion algorithm, which assumes that the evidence is given temporally-uniform decision weights, and the leaky competing accumulator model (LCA), which predicts a variety of temporal weighting patterns, including (for some model parameters) a specific interaction between stimulus duration and temporal weighting. While the LCA-predicted interaction was confirmed in some of the observers (who performed multiple sessions with the moving dots displays), future work will be needed to further characterize how temporal weighting of evidence depends on task characteristics and individual differences. The issue of temporal weighting and its dependence on characteristics of evidence accumulation and type of decision-boundary is further discussed in a review paper by Zhang (2012), who also examines how these characteristics affect decision optimality. Lastly, two papers discuss the neural mechanisms of perceptual decisions. Simen (2012) examines a two-layer neural model that includes accumulators and bistable cell-assemblies that can implement the decision-boundary—which is assumed without much discussion in the standard approach—and discusses the difficulties of mapping those processing units to the neural recordings observed in brain data. van Vugt et al. (2012) use a model-driven approach to reveal the EEG correlates of evidence accumulation for a motion discrimination task. The authors use a novel computational technique to show that the time-course of the EEG activity demonstrated a non-linear profile—a finding that may arbitrate the dispute between linear (e.g., Brown and Heathcote, 2008) and non-linear (e.g., Usher and Mcclelland, 2001) models of evidence integration. Moreover, this paper indicates the possibility of identifying individual differences in evidence integration (e.g., speed-accuracy trade-off) from the EEG signal, offering a useful tool for characterizing the computational properties of the decision mechanism.

## Adaptive decision making

The second group of six articles examines decisions that extend over a longer time-frame and which allow the subject to control the evidence accumulation process, and to form and update beliefs about the state of the environment. The study by Knox et al. (2011) of exploration and exploitation suggests that human decision makers learn from interaction with their environment in a reflective manner (without requiring direct observation of changes in the environment) but yet do not plan optimally because they do not consider the long-term information value of actions. The contribution by Osman and Speekenbrink (2012) extends this inquiry by studying how knowledge about the values of actions can be affected by tasks of prediction (outcome estimation) and control (interventions to achieve an outcome). They demonstrate a distinction between prediction and control whereby controllers were able to transfer their knowledge to tests of prediction but not vice versa. In this way, the concept of control is similar to that of planning for a goal rather than for adapting to an environment as in Knox et al. (2011) but, in both of these papers, decision makers cycle from evidence accumulation, to action, to feedback, and back again (cf. model-based learning; Sutton and Barto, 1998). Yu and Lagnado (2012) use this framework in a slot machine paradigm to show that, while participants over time came to understand the observed environment (slot machine payouts) accurately, their understanding of the underlying structure of the environment was flawed. Beliefs about structure and causality were more strongly influenced by initial beliefs than by experience. Also studying decisions from experience, Dutt and Gonzalez (2012) explore the role of inertia, or the tendency to repeat one's final decision, irrespective of its outcome. They show both the advantages and disadvantages of incorporating inertia into an instance-based learning model of repeated binary choice. In contrast to this focus on inertia, Lange et al. (2012) demonstrate how decision makers adapt to the environment, using a new model that combines the HyGene model (Thomas et al., 2008) with the context-activation model (Davelaar et al., 2005). Across two experiments that manipulate serial order, consistency of newly-acquired evidence with previously-generated hypotheses, and elicitation timing, the authors show that not all data have an equal impact on hypothesis generation processes: newly-acquired data can cause inconsistent hypotheses to be purged from working memory. The authors propose that whether this results in a recency or primacy effect is likely to depend on the richness of the information and its rate of presentation.

## Preference-based decisions

The next group of three articles addresses preference formation, in situations (risky choice and multi-attribute decisions) that do not set up an objective/normative criterion, but rather leaves this to the subject's control. Fiedler and Glockner (2012) monitor how people choose between lotteries using eye-tracking to distinguish between competing models of risky choice. The results disconfirmed Take-the-Best, or lexicographic heuristics, in favor of compensatory models that assume observers integrate outcomes with attentional weights determined by outcome probability. In particular, people gather more information within (rather than between) lotteries and they tend to gather more information (toward the end of the decision) from the chosen alternative, indicating top–down feedback from alternative to processing representations. Also using eye-tracking, Krajbich et al. (2012) propose a formalization of the influence of visual fixations on the dynamics of preference formation. The authors build on the attentional diffusion model (aDDM), which modulates the rate of evidence-accumulation depending on the position of visual fixation, to explain the responses and reaction times of human subjects during purchasing (accept/reject) decisions. The study demonstrates how small attentional fluctuations during the deliberation period can influence the decision outcome. This approach is closely related to theoretical models of multi-attribute choice [e.g., decision field theory, Roe et al. (2001); and value-based LCA, Usher and Mcclelland (2004)], in which attentional switching to different choice aspects drives preference formation. This class of models is extended in Wollschlager and Diederich (2012), which presents a novel model of contextual preference reversal (attraction, similarity, and compromise effects) for multi-alternative, multi-attribute choice: the 2N-ary Choice Tree model. The model offers closed-form expressions for choice probabilities and response time distributions and, contrary to previous theories, explains reversal effects by assuming that attentional weights depend on the alternatives in the choice-set [cf. a recent study, which appeared after this Research Topic and provides an explicit mechanism for how the alternatives affect weights to the choice attributes: Bhatia (2013)].

## Novel or integrative approaches

Finally, two papers aim to provide novel or integrative frameworks for understanding dynamical decision making. Trueblood and Busemeyer (2012) present a decision model based on principles of quantum theory—a radical shift from the standard framework—which provides a novel account of order effects in belief updating and inference. This paper provides an introduction to the elegant principles of quantum probability. This theory is of great potential, although stronger data might be needed to persuade the skeptical readers (e.g., showing cyclic changes in order effects). In the last paper Fox et al. (2013) present an overarching framework for the entire decision making cycle, from the framing of a decision to establishing preferences and making commitments. They extend the standard model to more ecological and dynamic situations, in which the alternatives are not predefined and the agent faces a variety of constraints and conflicts. The theory situates dynamical decision making with respect to other high-level cognitive capabilities such as problem solving, planning and collaborative decision-making.

## Conclusions and future work

We believe that this collection has revealed a number of important aspects of the nature of decision processes. More importantly, we hope that it will stimulate readers to keep probing these processes. Various key questions are still unresolved. How close are people to optimality when making decisions? Why does this vary so much between the cases of evidence and preference? Is the Bayesian framework a general one for all types of decisions (can one extend it to more complex cases that allow the subject control over the information flow and the decision criteria?). What are the neural mechanisms, and the nature of individual differences? Future research into these topics, should surely keep us stimulated for the near future.
